# Blunt Pancreatic Injury in Major Trauma: Decision-Making between Nonoperative and Operative Treatment

**DOI:** 10.1155/2018/6197261

**Published:** 2018-02-22

**Authors:** Christopher Ull, Sebastian Bensch, Thomas Armin Schildhauer, Justyna Swol

**Affiliations:** ^1^Department of General and Trauma Surgery, BG University Hospital Bergmannsheil, Bürkle-de-la-Camp-Platz 1, 44789 Bochum, Germany; ^2^Department of Pulmonology, Intensive Care Medicine, Paracelsus Medical University, Nuremberg, Germany

## Abstract

Blunt trauma injuries to the pancreas are rare but are associated with significant overall mortality and a high complication rate. Motor vehicle collisions are the leading cause of blunt pancreatic trauma, followed by falls, and sports injuries. We discuss the decision-making process used during the clinical courses of 3 patients with life-threatening blunt pancreatic injuries caused by traumatic falls. We also discuss the utility of the American Association for the Surgery of Trauma Organ Injury Scale (AAST-OIS), which provides a system for grading pancreatic trauma. Retrospectively, the cases reviewed were classified as AAST-OIS grade II, III, and IV in each one patient. Although the nonoperative approach was initially preferred, surgery was required in each case due to pseudocyst formation, pancreatic necrosis, and posttraumatic pancreatitis. In each case, complete healing was achieved through exploratory laparotomy with extensive lavage and placement of abdominal drains for several weeks postoperatively. These cases show that nonoperative management of pancreatic ductal trauma results in poor outcomes when initial therapy is less than optimal.

## 1. Background and Case Series Presentation

Blunt pancreatic injuries are usually the result of a substantial force to the upper abdomen that results in compression of the pancreas against the lumbar vertebrae [1]. Car accidents are the predominant cause of such injuries, followed by falls, and sports injuries [2]. Approximately half of pancreatic lesions occur in the setting of multiple injuries caused by a major trauma. Injuries to the pancreas are generally rare because of its retroperitoneal location, which offers relative protection to the gland. Currently, pancreatic injuries account for less than 2% of blunt abdominal trauma injuries [3, 4]. However, pancreatic trauma is associated with significant overall mortality and a complication rate of more than 60% [5, 6]. Frequent complications after blunt pancreatic trauma include fistula and pseudocyst formation, traumatic pancreatitis and pancreatic abscesses, and less frequent complications such as peritonitis, gastrointestinal bleeding, and splenic vein thrombosis [2, 7]. The most widely accepted classification and grading system for pancreatic trauma is the Organ Injury Scale (OIS) developed by the American Association for the Surgery of Trauma (AAST) [8]. The main determinants of the score using this scale are the location of the injury and the presence of damage to the pancreatic parenchyma and the ductal system (Table 1).

We retrospectively report the diagnoses, management, and subsequent clinical courses of three patients with life-threatening blunt pancreatic injuries caused by traumatic falls. All these patients received emergency whole-body CT at presentation. Although the nonoperative or radiological (CT-guided intervention) approach was initially preferred, surgery was ultimately required in each case due to pseudocyst formation, pancreatic necrosis, and posttraumatic pancreatitis. In each case, complete healing was achieved through exploratory laparotomy with extensive lavage and the placement of abdominal drains for several weeks postoperatively.

### 1.1. Patient 1

**Figure 1:**
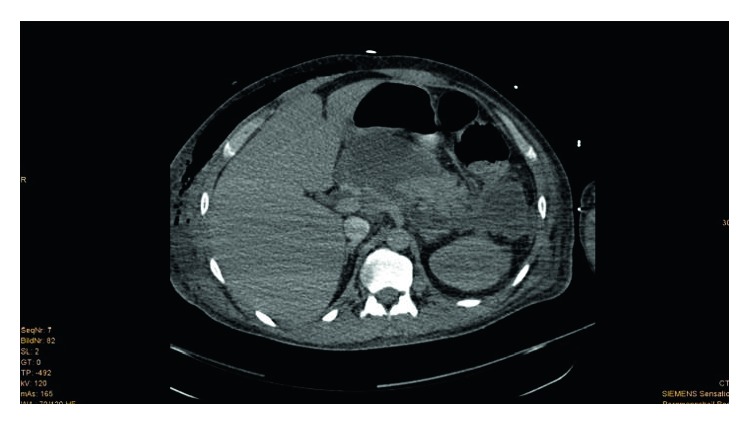
(Patient 1, scan 1) 18 days after trauma. CT scan of the abdomen showing pancreatitis with large expanding pseudocysts with a maximum diameter of 8 cm in the pancreas head-corpus transition area. Pigtail drainage was placed with guidance from CT in the fluid accumulation/pseudocyst from the left flank side. The drainage system emptied brown-tinged serous fluid.

A 31-year-old male fell from an unknown height at the scene of a crime. Injuries diagnosed in the primary survey included severe brain trauma, facial fractures, serial rib fractures, bilateral lung contusions, a right-sided hemopneumothorax, bilateral pelvic fractures, intraperitoneal bladder rupture with urethral rupture of the membranous urethra, a distal femoral fracture, and a fracture of the right 5th metatarsal, resulting in an ISS of 41. During the primary survey, the pancreatic lesion was not detectable. During the first 5 days of the clinical course, acute respiratory failure occurred. The condition of the patient required cannulation for venovenous extracorporeal membrane oxygenation (VV-ECMO). Weaning from VV-ECMO and from the invasive ventilation was subsequently successful, indicative of pulmonary recovery. Then, clinical findings of deterioration arose, including increased inflammatory markers. CT scan showed massive pancreatitis with formation of a 6 cm pseudocyst at the transition from the body to the head and extensive fatty tissue necrosis in the tail of the pancreas (Figure 1). The ductal injury was presumed in this case, but it was not detected on CT scan. We tried to minimize the interventions to this patient after he suffered severe ARDS treated with ECMO. For this reason, surgical decision-making indicated CT-guided drainage as first therapy attempt.

**Figure 2:**
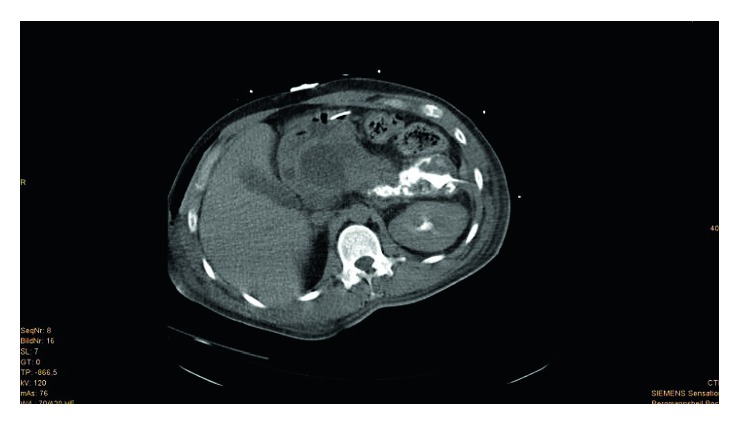
(Patient 1, scan 2) CT scan 25 days after trauma. After iatrogenic removal of drainage, a large retrogastric pseudocyst with a surrounding capsule was observed. Obviously, a narrow aisle was found where fluid accumulation continued to expand caudally below the transverse column. The pseudocyst was again punctured from the left lateral side with 9.5 French Lunderquist drainage, and contrast medium was injected into the pseudocyst cavity. There was a narrow connection path for retrogastric fluid accumulation. Thus, all the caves were drained adequately.

The cyst in the pancreas was successfully drained using guided CT (Figure 2). Despite successful CT-guided drainage of the cyst, a new cyst developed, along with ascites and elevated laboratory markers, indicative of infection. Operative treatment was indicated at that time due to the failure of interventional drainage treatment. Exploratory laparotomy and intraperitoneal lavage were performed, and a drainage remained in situ. Intraoperatively, inflammation of the whole small intestine was discovered, likely caused by rupture of a pancreatic pseudocyst with leakage of pancreatic secretions into the peritoneum.

**Figure 3:**
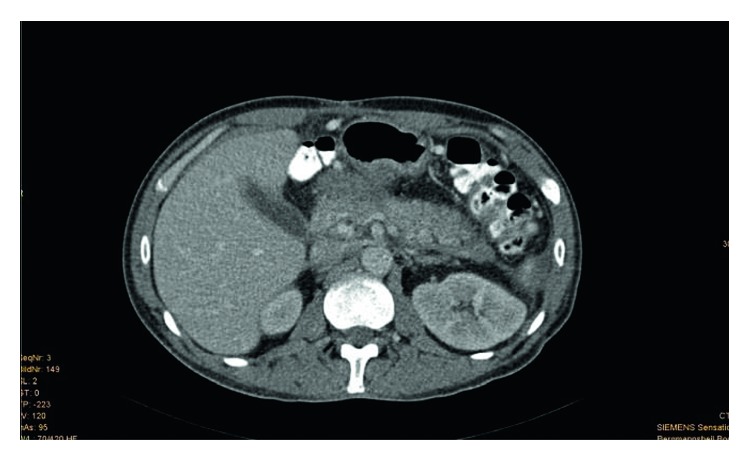
(Patient 1, scan 3) CT scan 4 months after trauma. Compared to the previous CT scan, no additional evidence of new pseudocyst or abscess formation was found. The drains that were previously placed in the bursa omentalis were removed.

Subsequently the pancreatic injury was classified during the surgery as a grade III laceration due to the AAST-OIS. The drain was removed six weeks postoperatively. No recurrent symptoms appeared (Figure 3). The patient was discharged from the hospital four months after the accident with disabilities due to the injury of the lower extremity.

### 1.2. Patient 2

**Figure 4:**
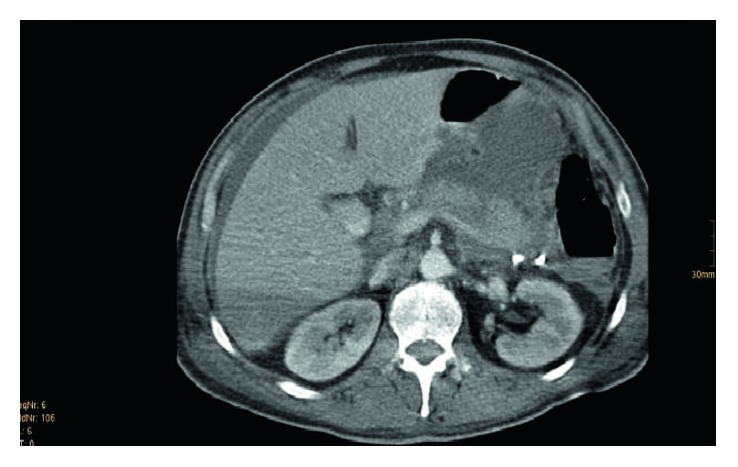
(Patient 2, scan 1) CT scan 15 days after trauma and admission after hospital referral. CT scan after the introduction of drainage from the left lateral to the retroperitoneal. The pancreas appears to be altered beside the corpus and the tail. In particular, hypointense formation in the pancreatic body is shown in the ventral part of the pancreas due to the injury. It connects with the remaining drainage. Free fluid is shown in the perihepatic and perisplenic areas.

**Figure 5:**
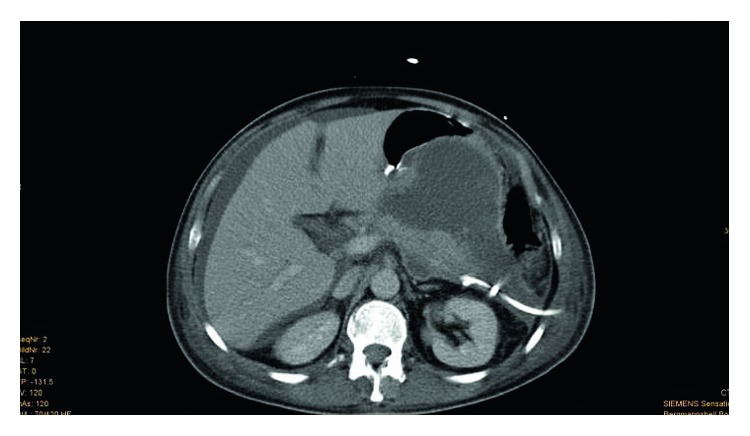
(Patient 2, scan 2) CT scan 22 days after trauma. Compared to the CT scan 7 days ago, there was an increase in septate fluid accumulation in the bursa omentalis and peripancreatically in the demarcation of the Gerota fascia. Drainage from the left side was still present. In the case of known pancreatic injury, the pancreas has a linear (sometimes up to 1 cm subtotal) interruption of the parenchyma, as in the case of subtotal rupture in the distal third of the pancreas corpus. The other parts of the pancreas were homogeneously contrasted. There was a significant increase in perihepatic ascites and no abscesses.

A 50-year-old male fell approximately 20 meters from the basket of a hot air balloon. The following injuries were diagnosed in the primary survey: fractures of the sixth through eighth and eleventh thoracic vertebrae, complete paraplegia below T7, a fracture of the first lumbar vertebra, serial rib fractures, a lung contusion, a right-sided hemopneumothorax, and pancreatic injury. The ISS was 25. The pancreatic lesion was detected in the primary survey and classified according to the AAST-OIS as a grade II laceration. The initial treatment intervention was CT-guided drainage, which was performed at a referral hospital (Figure 4). ERCP showed no leakage or rupture of the pancreatic duct. Two weeks after the trauma, the patient was stable for transport and was admitted to the study ICU, which specializes in the early rehabilitation of spinal cord injuries. Increased inflammatory markers were seen at the beginning of the third week after admission. Methicillin-resistant *Staphylococcus aureus* (MRSA) was detected in the abdominal secretions. CT scan showed a pancreatic fistula, and the pancreas showed altered signal with a hypointense formation found in the pancreatic body, indicating pancreatic injury. There was a blockage of peripancreatic fluid despite the in situ drain (Figure 5). Because nonoperative treatment failed, an exploratory laparotomy was performed. During surgery, most of the pancreas was found to be necrotic; necrosectomy with partial pancreatectomy and abdominal lavage and drain placement were performed. The drain was removed seven weeks postoperatively.

Further treatment was complicated by a massive pulmonary embolism, which was lysed successfully. The patient was discharged from the hospital after completing rehabilitation for his spinal cord injury.

### 1.3. Patient 3

**Figure 6:**
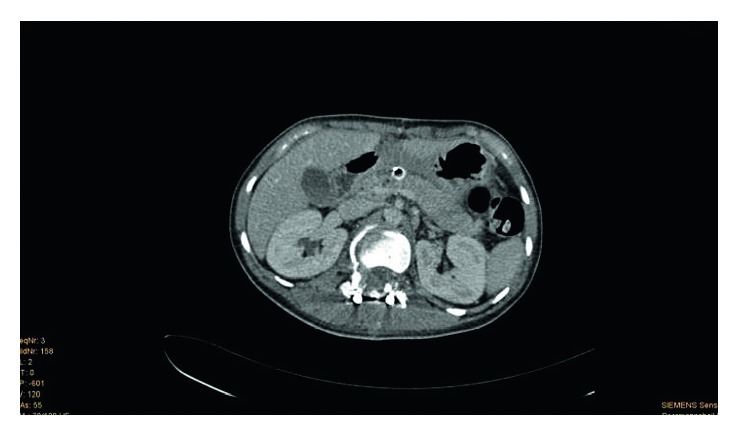
(Patient 3, scan 1) CT scan 19 days after trauma and 17 days after laparotomy due to pancreatitis. Drainage at the level of the upper abdomen placed along the left hepatic lobe of the pancreas head and body. There are discrete air pockets between the left hepatic lobe and the pancreatic head. The pancreas appears to be vigorous and slightly blurred. In the peripancreatic tissue, low-density increases are observed. No new liquid formations are observed.

A 25-year-old female attempted suicide and fell from approximately 7 meters. The following injuries were diagnosed in the primary survey: fractures of the first and second lumbar vertebrae, incomplete paraplegia below L2, a type III open fracture of the right lower extremity, a fracture of the left calcaneus and a pancreatic contusion. These injuries resulted in an ISS of 26. Pancreas contusion was detected in the primary survey trauma scan. Within the first three days of hospitalization, the patient developed an acute abdomen due to peritonitis. Urgent laparotomy, lavage and abdominal drain placement were conducted. Intraoperatively, the head of the pancreas was considerably rotated, causing significant bleeding, and the lesser sac was filled with small vessel bleeding. Macroscopic rupture was not present. The injury was classified using the AAST-OIS as a grade IV laceration. Postoperatively, CT findings showed indistinguishable pancreatic tissue. Recurrent clinical deterioration occurred with symptoms including abdominal pain and increased inflammatory markers (Figure 6). The infection was treated successfully with eight weeks of antibiotic therapy and consistent flushing of the drain. The patient was discharged from the hospital with paraplegia after 3 months of rehabilitation for her spinal cord injury.

## 2. Discussion

Falls from various heights were responsible for pancreatic injury in each of the cases in this series. The diagnoses were made using a combination of clinical observations, laboratory markers, ultrasonography, and CT imaging. Retrospectively, the blunt trauma to the pancreas was classified as an AAST grade II, III, or IV injury in each case (Table 2). Surgical treatment was necessary during hospitalization in each case due to common complications of pancreatic trauma [7] that occurred independently of the AAST-OIS grades. Two patients developed pancreatic pseudocyst and necrosis despite prior nonoperative treatment with drains placed under CT guidance. The third patient developed peritonitis due to posttraumatic pancreatitis. In all cases, complete healing was achieved after exploratory laparotomy with extensive lavage and placement of in situ abdominal drains for several weeks. Consistent flushing of the drain and antibiotic therapy are necessary during the postoperative period. The choice of the antibiotic depends on the hospital antibiogram but should include agents like carbapenems that provide broad spectrum coverage including coverage of gram-negative agents. Carbapenems are suited for this purpose.

### 2.1. Decision-Making

Grading of pancreatic injuries is essential for determining the course of treatment. Nonsurgical treatments are common with grade I and grade II injuries, whereas the involvement of the pancreatic duct in a grade III and IV or more severe injury usually requires surgical treatment [2]. Kao et al. reported both an increased rate of complications and increased mortality rate with each increase in the AAST grade of pancreatic injury [5]. This finding was confirmed in the ReCONECT study by Velmahos et al., which included 230 patients with pancreaticoduodenal injuries [9]. A significant correlation between the AAST pancreatic injury grade and complications was also observed by Krige et al. [10]. However, in this study, a significantly greater number of patients with AAST grade I and grade II injuries died compared with patients with higher-grade injuries. Overall, the progressive destruction of the pancreatic parenchyma leads to a higher rate of complications, but the relationship between the AAST grade and mortality has not been clarified in detail.

### 2.2. Learning Points

Relying on laboratory tests and conservative approaches to treatment may overlook and delay the appropriate management of blunt pancreatic trauma of ASST-OIS grades II–V. Early pancreatic injury often appears subtile on computed tomography imaging (Table 2). Because pancreatic trauma can exhibit temporal evolution, serial imaging with CT or MRI is important in management, as demonstrated by the 3 cases we described.

Although surgery is the preferred treatment for grade III and IV pancreatic trauma, nonoperative management is increasingly being recommended for blunt pancreatic trauma [11, 12]. In hemodynamically stable patients with grade III injury and a controlled leak that is walled off as a pseudocyst without associated organ injuries and pancreatic necrosis, nonoperative management is preferred. Posttraumatic and necrotizing pancreatitis is associated with nonoperative procedure failure and indication for operative proceedings.

Nonoperative management of pancreatic trauma is on the rise [11, 12]. Endoscopic and percutaneous techniques for pseudocysts and pancreatic necrosis have a high success rate and can prevent the need for open or laparoscopic necrosectomy at times. Pancreatitis that is associated with necrosis, hemorrhage, abscess, pseudocysts, enteric fistulae, and organ failure limits the physiological reserve and warrants special care.

These cases are examples of what happens when initial therapy is less than optimal for these patients and show that nonoperative management of pancreatic ductal trauma results in poor outcomes. The issue is whether or not the duct is injured. If there is just bruising to the body of the pancreas, then nonoperative management is possible. If there is ductal disruption, ongoing egress of pancreatic fluid will result in diffuse saponification and pancreatitis. For this reason, surgery is recommended in similar situations [11, 12].

## Figures and Tables

**Table 1:** American Association of the Surgery of Trauma classification of pancreatic trauma-Organ Injury Scale (AAST-OIS) [8].

Grade	Injury	Description of the pancreatic injury
I	Hematoma	Minor contusion without ductal injury
	Laceration	Superficial laceration without ductal injury
II	Hematoma	Major contusion without ductal injury or tissue loss
	Laceration	Major laceration without ductal injury or tissue loss
III	Laceration	Distal transection or pancreatic parenchymal injury with ductal injury
IV	Laceration	Proximal transection or pancreatic parenchymal injury involving the ampulla
V	Laceration	Massive disruption of the pancreatic head

**Table 2:** Comparison of grade of injury due to American Association of the Surgery of Trauma classification of pancreatic trauma-Organ Injury Scale (AAST-OIS) [8].

Patient	Grade of injury due to AAST-OIS classification of pancreatic trauma [8]		
	At presentation	Prelaparotomy/CT scan	Intraoperatively
1	Missed injury	Grade II/ductal injury presumed	Grade III
2	Grade II	Grade II/deterioration to grade III	Grade III
3	Grade I	Ductal injury presumed due to acute abdomen symptoms, no CT scan, emergency laparotomy	Grade IV

## Abbreviations

AAST: American Association for the Surgery of Trauma
ECMO: Extracorporeal membrane oxygenation
CT: Computed tomography
ISS: Injury Severity Score
ICU: Intensive care unit
OIS: Organ Injury Scale.
